# Tunable optical anisotropy in epitaxial phase-change VO_2_ thin films

**DOI:** 10.1515/nanoph-2022-0153

**Published:** 2022-06-01

**Authors:** Jimmy John, Amine Slassi, Jianing Sun, Yifei Sun, Romain Bachelet, José Pénuelas, Guillaume Saint-Girons, Régis Orobtchouk, Shriram Ramanathan, Arrigo Calzolari, Sébastien Cueff

**Affiliations:** Université de Lyon, Institut des Nanotechnologies de Lyon (INL) UMR 5270 CNRS, École Centrale de Lyon, 36 Avenue Guy de Collongue, Ecully 69134, France; CNR-NANO Istituto Nanoscienze, Modena I-41125, Italy; J. A. Woollam, Co., 645 M Street, Suite 102, Lincoln 68508, NE, USA; School of Materials Engineering, Purdue University, West Lafayette 47907, IN, USA

**Keywords:** optical anisotropy; phase-change materials, tunable nanophotonics, vanadium dioxide

## Abstract

We theoretically and experimentally demonstrate a strong and tunable optical anisotropy in epitaxially-grown VO_2_ thin films. Using a combination of temperature-dependent X-ray diffraction, spectroscopic ellipsometry measurements and first-principle calculations, we reveal that these VO_2_ thin films present an ultra-large birefringence (Δ*n* > 0.9). Furthermore, leveraging the insulator-to-metal transition of VO_2_, we demonstrate a dynamic reconfiguration of optical properties from birefringent to hyperbolic, which are two distinctive regimes of anisotropy. Such a naturally birefringent and dynamically switchable platform paves the way for multi-functional devices exploiting tunable anisotropy and hyperbolic dispersion.

## 1 Introduction

Optical anisotropy has been a subject of fundamental interest for decades and finds applications in many different areas, ranging from standard optics – such as beam splitters, polarizers, waveplates and modulators – to displays, medical analysis and sensing. The fundamental origin of anisotropies in atomic, molecular or optical systems is intrinsically linked to their underlying symmetries. For example, light propagating in a centrosymmetric cubic lattice of crystalline silicon sees the same refractive index in all directions, whereas the same optical wave impinging on an anisotropic crystal will see angle-dependent refractive indices. In other words, the structural asymmetry leads in this case to asymmetric optical properties. Although naturally birefringent bulk materials (e.g., quartz) were largely exploited in devices, the need for compactness and efficiency drove researchers to imagine artificial materials with large optical anisotropies. With recent progress in nanofabrication, the realization of asymmetric nanostructured materials enabled the demonstration of controlled optical anisotropies with desired functionalities [1–3].

Interestingly, combining such strongly anisotropic media with isotropic ones leads to intriguing physics and unconventional optical modes. For example both Dyakonov surface waves [4, 5] and the so-called relaxed total internal reflection [6] are low-loss optical modes supported by ultrathin films at the interface between anisotropic and isotropic media. Based on similar isotropic/anisotropic combinations, recent works demonstrated a quality factor enhancement in resonators [7] and anisotropy-induced photonic bound states in the continuum [8].

Although powerful, the use of nanostructured materials can severely limit the potential designs and architecture of future devices. On the other hand, the use of materials with naturally strong anisotropies would considerably ease the integration, and their subsequent patterning, opening new degrees of freedom in nanophotonic design [9]. There is therefore a renewed interest for the discovery of materials with naturally strong optical anisotropy. In that context, large optical anisotropies were recently reported in a quasi-one-dimensional crystal of BaTiS_3_ [10], in van der Walls heterostructures [11, 12], in Halide Perovskites [13, 14] and in transition-metal dichalcogenides [9, 15], [16], [17].

**Figure 1:**
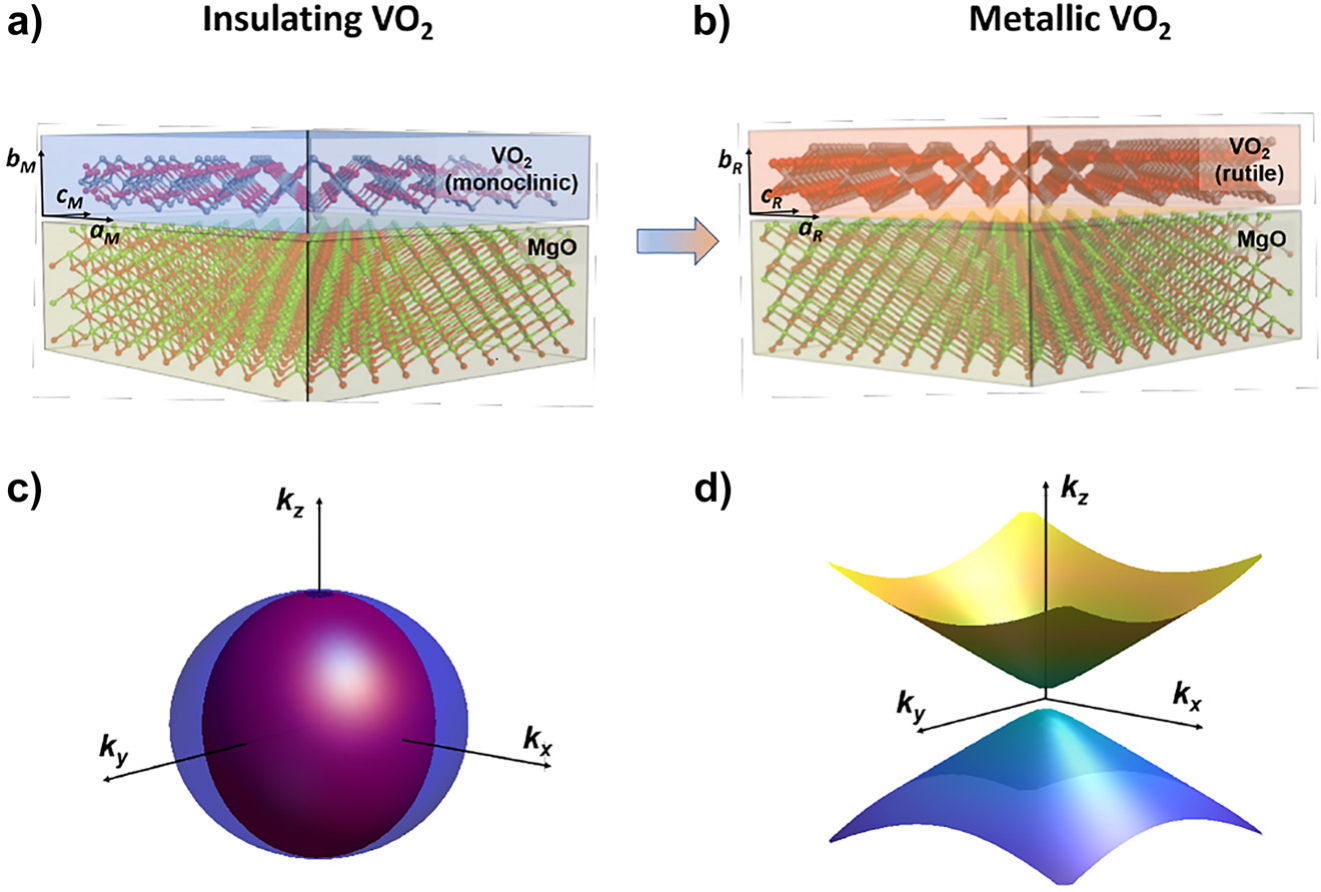
Structural and optical anisotropy in VO_2_ thin films. (a and b) Schematic illustrating the tunable anisotropy of epitaxial films of VO_2_. The crystallographic axes directions are set by the epitaxial relationship between the VO_2_ layer and the MgO substrate. The differences of lattice parameters between in-plane and out-of-plane directions produce a strong birefringence, as illustrated by the isofrequency contour in (c). (b) The insulator-to-metal transition modifies the lattice parameters and introduces free carriers. The combination of these effects leads to reconfigure the anisotropy from birefringent to hyperbolic in the near-infrared, as shown by the isofrequency contour at *λ* = 1 μm (d).

So far, these optically anisotropic materials are passive and their related applications remain static. Exploring ways to dynamically modulate such anisotropies would open up interesting opportunities for both fundamental and applied studies. Interestingly, bulk materials that are both anisotropic and tunable do exist. Among them, vanadium dioxide (VO_2_), presents a first-order insulator-to-metal transition resulting in strongly tunable physical properties, and crystallizes in a monoclinic – and thus biaxial – system [18–20]. VO_2_ therefore appears to be an excellent candidate for integrated tunable anisotropy. However, their monolithic integration as thin films usually results in isotropic polycrystalline thin films. Here, we demonstrate, both theoretically and experimentally, a strong and tunable optical anisotropy in epitaxially-grown VO_2_ thin-films. To do so, we selected a substrate with engineered lattice parameters and orientations to accommodate epitaxial growth of VO_2_ thin-films. Using a combination of X-ray diffraction and spectroscopic ellipsometry measurements, we experimentally confirm the epitaxial growth of VO_2_ on MgO (111), and demonstrate a large tunability of structural and optical anisotropy in VO_2_ thin-films that is confirmed by theoretical simulations. As illustrated in Figure 1, our study not only reveals an ultra-large birefringence for the monoclinic-insulating state, but also a switching to a hyperbolic dispersion regime upon triggering the insulator-to-metal transition of VO_2_. This unique combination of natural anisotropy and tunable hyperbolicity opens to previously unexplored multifunctional optical applications.

In the next section (Section 2), we focus on the fabrication and structural characterizations of the system, in order to understand in more details the atomic arrangement of the VO_2_ layer. We then describe in Section 3 the optical characterization and the method used to extract the different in-plane and out-of-plane optical components of the VO_2_ thin films. In Section 4 we provide a detailed atomistic analysis of the system using density functional theory calculations. Finally, we discuss the future implications of our findings in Section 5.

## 2 Fabrication and structural characterization

Bulk VO_2_ is known to crystallize in a monoclinic structure at room temperature, with the following lattice parameters: *a* = 5.75 Å, *b* = 4.52 Å and *c* = 5.38 Å. At temperatures higher than 341 K, VO_2_ transitions to a tetragonal rutile structure, with the modified lattice parameters *a* = 4.55 Å and *c* = 2.85 Å.

For the fabrication of thin films, we start from a (111) oriented MgO substrate, specifically chosen to accommodate epitaxial growth of VO_2_ [21]. Magnetron sputtering was used to fabricate the VO_2_ thin films using a V_2_O_5_ target. The optimized conditions to obtain high-quality thin-films were found to be as follows: growth temperature 650 °C, RF power output 100 W, atmosphere for deposition: 49 Ar/1 O_2_ with the flow rate of 100 ml per min and background pressure 5 mTorr. The growth rate for VO_2_ on MgO was estimated to be 40 nm per hour, resulting in a 70-nm-thick layer.

To understand in more details the growth, crystalline structure and symmetries of the fabricated VO_2_ thin films, we performed X-ray diffraction measurements with a Rigaku Smartlab diffractometer equipped with a rotating anode operating at 9 kW monochromatized with a two reflection Ge (220) crystal, which selects the Cu K*α*1 radiation (wavelength = 1.5406 Å). The detector was a scintillation counter.

**Figure 2:**
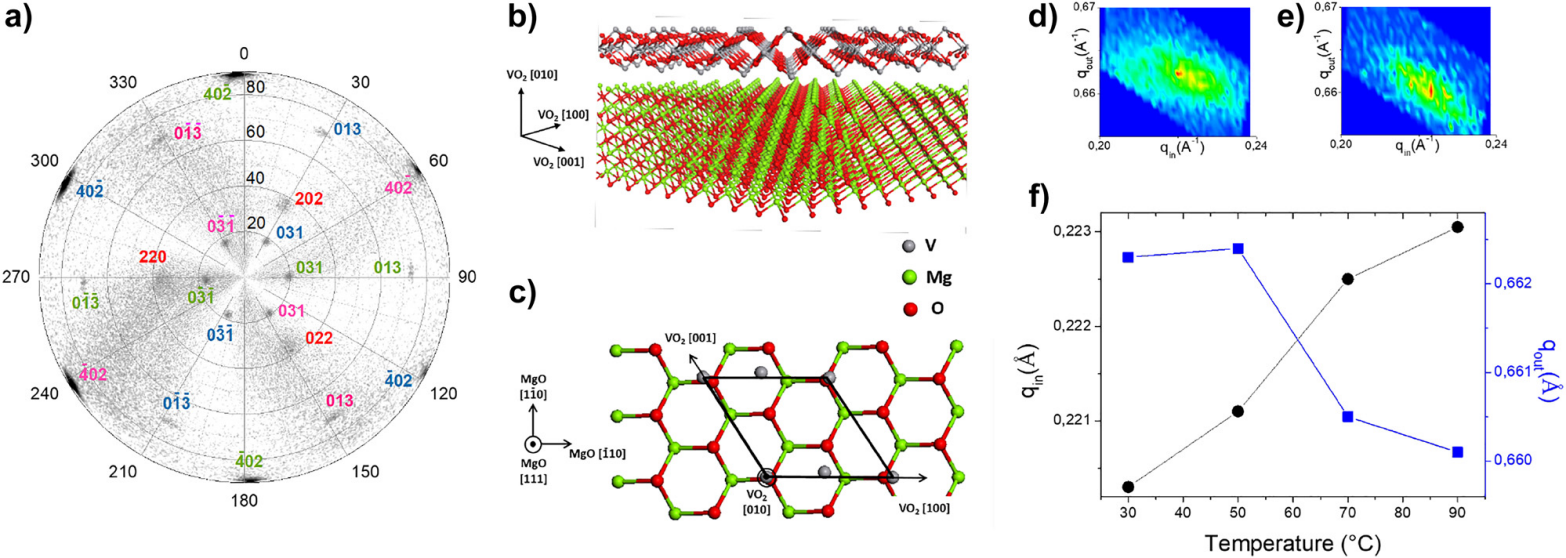
Structural characterizations in epitaxial VO_2_ thin films. (a) Pole figure recorded around the VO_2_ (013) reflexion at 2*θ* = 64.94°. The VO_2_ (40 $\bar{2}$ ) and MgO <220> reflexions (in red) are also detected. The reflexions corresponding to different VO_2_ variants are labeled using different colors. The overall 3D arrangement of atoms is sketched in (b), showing an epitaxial monoclinic VO_2_ crystal on MgO (111). In the out-of-plane direction (*z* direction), only the VO_2_ [010] component (also known as *b* parameter), is present. The in-plane components are mixture of [100] and [001] (*a* and *c* parameters), arranged in six variants (only one is depicted here for clarity). One of the variants is sketched in (c), where the black polygon indicates the VO_2_ surface unit cell. Reciprocal space mapping of the 031 Bragg reflection measured at (d) 30 °C and (e) 90 °C. The reciprocal space mapping is plotted as a function of *q*_in_ and *q*_out_, which represent the projection of the scattering vector along the out-of-plane and the in-plane directions of the sample, respectively. (f) Evolution of *q*_in_ and *q*_out_ as a function of the temperature.

Figure 2(a) shows the pole figure recorded around the VO_2_ (013) reflexion at 2*θ* = 64.94°. It presents well defined reflexions, indicating that VO_2_ is epitaxially grown on MgO. The pole figure corresponds to a VO_2_ orientation with respect to MgO, defined as {010}[100] VO_2_//(111) $<1\bar{1}0>$ MgO, as sketched in Figures 1 and 2(a), consistently with that reported in Ref. [21]. The (010) plane of the VO_2_ structure, containing the *a* and *c* axes, is nearly three-fold symmetric, with an angle between the *a* and *c* directions of 122.6°, to be compared to 120° for an actual threefold symmetric plane as MgO(111). This promotes the (010)VO_2_//(111)MgO arrangement and its equivalent, (0 $\bar{1}$ 0)//(111)MgO. Besides, the distance between two nearest neighbors along the <110> directions on the MgO(111) surface is 5.158 Å, reasonably close to the interatomic distance along the [100] direction of the VO_2_(010) surface, namely about 5.37 Å. This leads to an alignment of these directions in a quite robust epitaxial relationship, as attested by the discrete nature of the reflexions on the pole figure. However, due to the symmetries of both crystals, six VO_2_ variants are formed: the [100] in-plane direction of the (010) VO_2_ plane equivalently aligns along the three <110> directions of the MgO(111) surface, and so does the [100] in-plane direction of the (0 $\bar{1}$ 0) VO_2_ plane. Note that due to the limited crystalline order, the variants corresponding to (010)-oriented VO_2_ could not be distinguished from that corresponding to (0 $\bar{1}$ 0)-oriented VO_2_ in the pole figure of Figure 2(a).

This analysis therefore enables us to precisely define the structure of the epitaxially-grown VO_2_ layers, which is illustrated in Figure 2(b and c). Importantly, it shows that the out-of-plane component (*z* direction) is solely composed of the *b*-parameter of VO_2_ ([010] direction), whereas the in-plane component (*x*–*y* plane) comprises a mixture of *a* and *c* parameters, arranged in six different variants.

We further measured and analyzed the modifications of these lattice parameters upon the insulator-to-metal transition. We used a temperature cell adapted to our X-ray diffraction equipment and follow one of the diffraction peak of VO_2_ as a function of temperature. Figure 2(d) shows the reciprocal space mapping of a 031 Bragg reflection measured at 30 °C. The sample was aligned in agreement with the following epitaxial relationship: 010[100]VO_2_//(111) $<1\bar{1}0>$ MgO. From these measurements the *b* and *c* lattice parameters are estimated to be 4.53 Å and 5.39 Å, respectively, in good agreement with the bulk monoclinic phase. The sample temperature was then increased by steps of 20 °C with a ramp of 30 °C/min and four reciprocal space mapping measurements were performed from 30 °C to 90 °C. Figure 2(f) shows the evolution of the center of the Bragg spot as a function of temperature. At 70 °C the diffraction spot is shifted in agreement with the monoclinic to tetragonal phase transition which is expected to occur at 68 °C for bulk VO_2_. Such large modifications of the lattice parameters should therefore result in substantial modulations of the physical properties, as discussed in the next sections.

## 3 Optical characterization

Spectroscopic ellipsometry has been increasingly adopted to characterize the VO_2_ optical behavior during film deposition, through phase transition or at post treatment [22–26]. VO_2_ presents broad absorptions at both insulator and metallic phases and poses challenges for optical analysis. Dynamic ellipsometry on thermally-induced transition and *in-situ* real-time spectroscopic ellipsometry have been demonstrated to successfully break the thickness-index correlation and to reliably determine the film thickness and the refractive index of the absorbing VO_2_ [23, 26]. Although their crystalline structures are known to present anisotropy, most works on VO_2_ thin-films report isotropic optical models either because of the lack of sensitivity to the out-of-plane refractive indices, or because of the polycrystalline nature of VO_2_ deposited on substrates without any lattice-matched orientations. In this work, we combine ellipsometry and transmittance measurements to demonstrate the optically anisotropic nature of epitaxially-grown VO_2_ thin-films.

The VO_2_ thin films were measured using a spectroscopic ellipsometer with dual rotating compensators covering wavelengths from 190 to 1700 nm (J. A. Woollam RC2). Mueller matrix data (16 components, including M11) were collected at 25 °C and 100 °C from 20° to 55° of angle incidence. The Mueller matrix ellipsometry employs two compensators that continuously rotates at different frequencies to create phase shifts between *p* and *s* polarized lights, thus moderating the polarization states. As a result, the technique enables resolving the full polarization response of a system as described by the 4 × 4 Mueller matrix containing 16 elements. We therefore measure the complete response of the sample to arbitrary states of polarization, including linear, circular and elliptical polarization states. In addition, transmission intensity spectra were collected at normal incidence.

A three-layer model, comprising from bottom to top a MgO substrate, a VO_2_ film and a roughness top layer, was used to describe the sample. Refractive index of MgO was pre-determined from a bare substrate. Kramers–Kronig consistent oscillators were used to describe the dielectric functions of VO_2_ films in their different states. The insulating state of VO_2_ was modeled by four Gaussian oscillators, whereas the metallic VO_2_ was modeled by two Gaussian oscillators, one Lorentz oscillator and an additional Drude model. An effective medium approximation (EMA) composed of the underlying VO_2_ and air was used to model the less dense layer on the surface (more information on the ellipsometry measurements and fits in the SI). This three-layer model enables us to decouple the optical properties of the thin-film from that of the substrate and to extract the intrinsic optical dispersion of the VO_2_ layer.

In order to unambiguously extract the correct optical dispersions of the thin films, a multi-fit procedure has been followed, where all measurements collected at all the different incident angles were fitted simultaneously with a same model. To further improve the sensitivity to the in-plane refractive index, we included in the analysis the transmission spectra. The latter are only affected by the optical properties of the in-plane component. When combined with ellipsometry, transmittance helps to isolate the film optical responses in the different directions and thus would unravel the potential anisotropy of the sample. This procedure was followed to calculate the in-plane and out-of-plane optical characteristics of the thin film separately.

The VO_2_ film was first assumed to be isotropic. Such a crude model is not able to simultaneously provide a satisfying match to both the Mueller matrix ellipsometry and the transmittance data. This outcome was expected, since an isotropic material would result in a Mueller matrix containing mainly diagonal elements. Here, the values and signs taken by the off-diagonal elements of the Mueller matrix are typical of an anisotropic system. More specifically, we measured significant M_12_ (M_21_) elements that express linear extinctions (i.e., the dichroism), and significant M_34_ (M_43_) elements that describe the linear retardance (i.e., the birefringence, see the full Mueller matrix in SI). According to the previous structural characterizations, we set VO_2_ to be uniaxially anisotropic, with different in-plane and out-of-plane refractive indices. This anisotropic model significantly improves the fits to both data sets: the mean square error (MSE), with respect to the experimental data is reduced by up to 40%.

**Figure 3:**
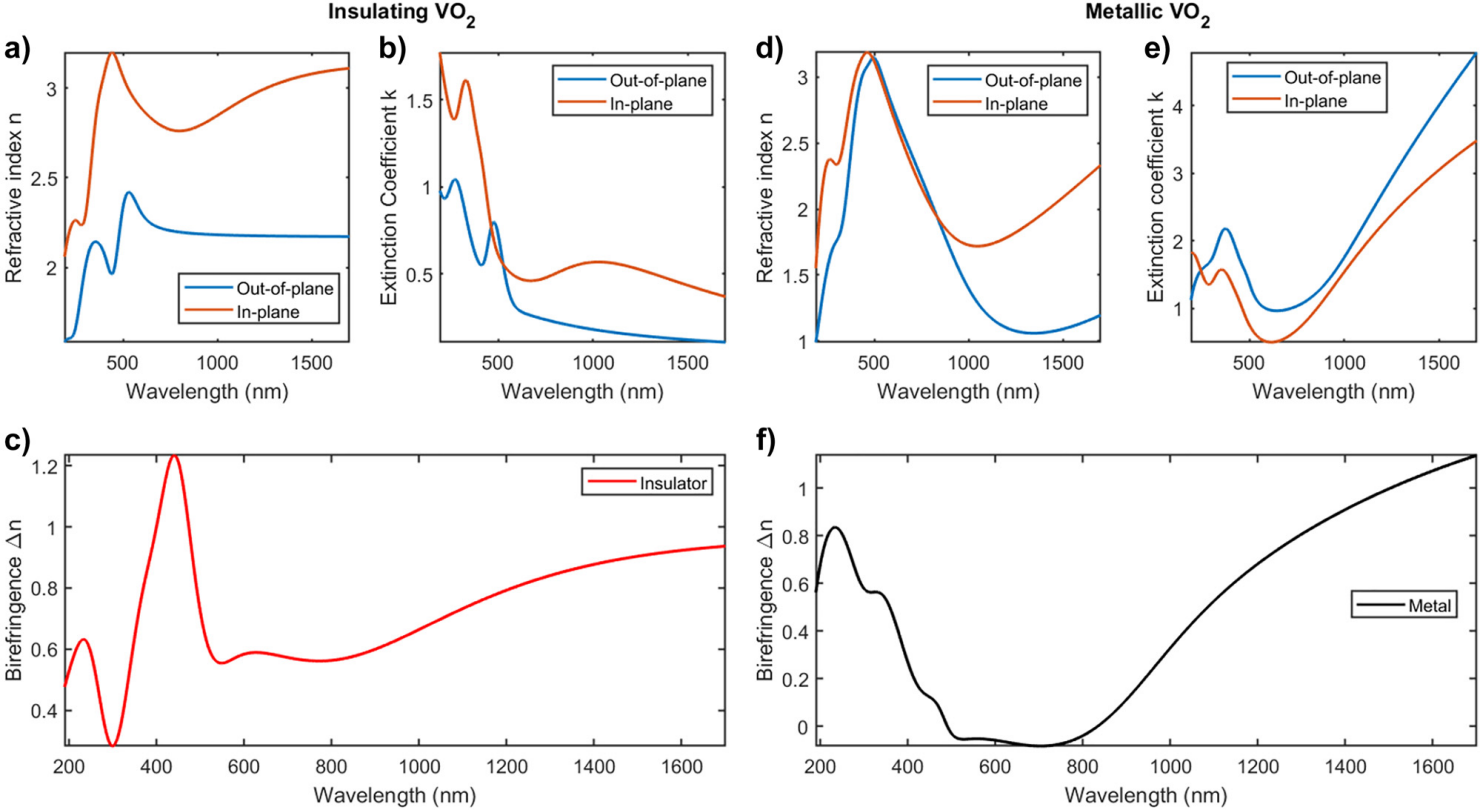
Optical properties of epitaxial VO_2_ thin films, extracted from spectroscopic ellipsometry in the range 190–1700 nm. In (a and b) and (d and e), both the in-plane (red lines) and out-of-plane (blue lines) complex refractive indices are plotted for the VO_2_ in its monoclinic insulating state (a and b) and for rutile metallic VO_2_ (b and c). (c and f) report the calculated birefringence values Δ*n* for both VO_2_ states, revealing a very large optical anisotropy throughout the measured wavelength range.

Therefore, combining Mueller matrix and transmittance measurements, it is possible to obtain the separate in-plane and out-of-plane contributions to the optical properties of VO_2_. As displayed in Figure 3, this analysis reveals a large optical anisotropy in the VO_2_ thin films, throughout the measured wavelength range. More specifically, a birefringence Δ*n* > 0.9 is measured in the near-infrared, that can fairly be considered as an ultra-large birefringence, as this value is higher than the recently reported giant optical anisotropy in BaTiS_3_ [10].

## 4 First principles simulations

In order to gain insights on the anisotropy characteristics described above, we carried out an extensive atomistic investigation of the structural, electronic, and optical properties of VO_2_ in the bulk and as deposited on MgO substrate. All simulations were carried out using first principles approaches based on density functional theory (DFT), as implemented in the Quantum-Espresso (QE) package [27]. The optical properties are evaluated starting from the calculation of the complex dielectric function $\hat{\epsilon }\left(\omega \right)=\epsilon_{r}+i\epsilon_{r}$ , as a function of the frequency (*ω* = *E*/ℏ) of the incoming radiation. $\hat{\epsilon }$ was obtained using the *epsilon.x* code, also included in the Quantum-Espresso distribution, which implements a band-to-band single particle formulation of the Drude–Lorentz model [28]. The refractive index (*n*) and extinction coefficient (*k*) are straightforwardly obtained from the dielectric function, through the relations:

(1) $$\begin{array}{ll} n & =\left\{\frac{1}{2}\left[{\left(\epsilon_{1}^{2}+\epsilon_{2}^{2}\right)}^{1/2}\right.\right.{\left.\left.+\epsilon_{1}\right]\right\}}^{1/2}\\ k & =\left\{\frac{1}{2}\left[{\left(\epsilon_{1}^{2}+\epsilon_{2}^{2}\right)}^{1/2}\right.\right.{\left.\left.-\epsilon_{1}\right]\right\}}^{1/2}. \end{array}$$

The computational details, along with the summary of the electronic properties of the three isolated bulk systems, namely rocksalt MgO, rutile VO_2_ (R-VO_2_) and monoclinic VO_2_ (M1-VO_2_), are reported in the SI.

### 4.1 VO_2_ bulk

In order to have a direct comparison with the optical results of Section 2, we calculated the refractive index (*n*) and extinction coefficient (*k*) of the VO_2_ bulk, in both the monoclinic (M1) and rutile (R) crystalline phases. The assumption of the bulk as representative of the system described above is justified by the thickness of the deposited films, which allows us to neglect the effects of the interface.

**Figure 4:**
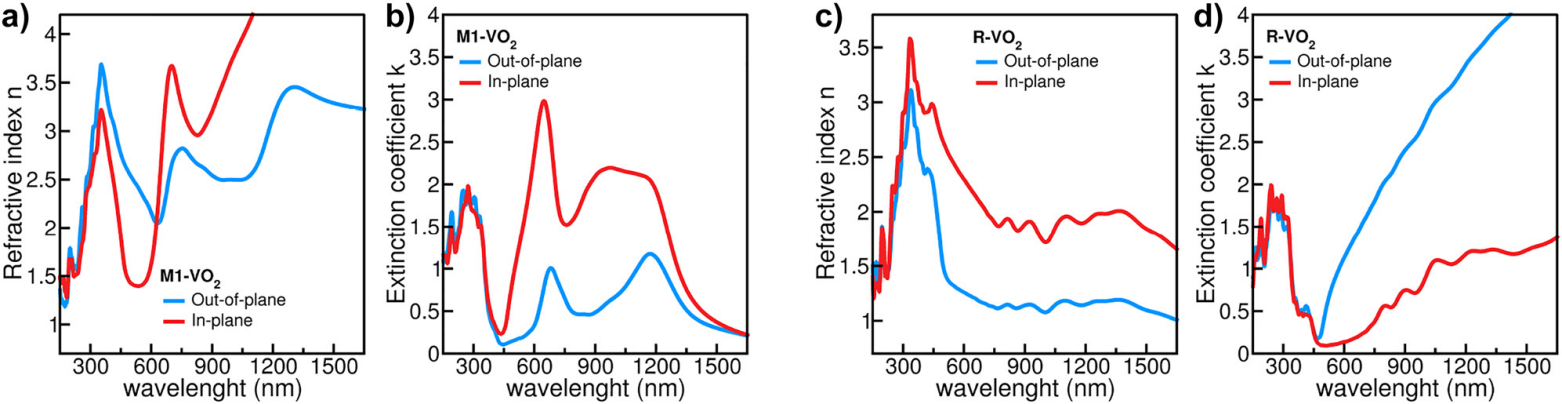
Simulated refractive index *n* (a and c) and extinction coefficient *k* (b and d) along the out-of-plane (blue line) and in-plane (red line) directions for VO_2_ crystalline bulk in the monoclinic (M1) and rutile (R) phase, as a function of the wavelength of incoming radiation.

Using the conclusion from the pole figure described in Figure 2, the bulk crystals have been oriented in space such that the *b* axis of both M1 and R structures is along *z* (i.e., out-of-plane, with respect to the film surface), while the *a* and *c* axes lie in the *xy* plane (i.e., in-plane). The results are shown in Figure 4.

The direct comparison with the results displayed in Figure 3 makes evident the excellent agreement between experiments and simulations. Indeed, it is possible to identify all the main spectroscopic features in the range 300–800 nm, as well as the main trends for higher wavelengths, for both the *n* and *k* values. This confirms the correct spatial orientation of the sample as in Figure 1, and unequivocally demonstrates the possibility to switch between the two VO_2_ phases even when the optical axis *c* lies in the film plane, and not in the perpendicular directions, as usually assumed.

### 4.2 MgO/VO_2_ interfaces

**Figure 5:**
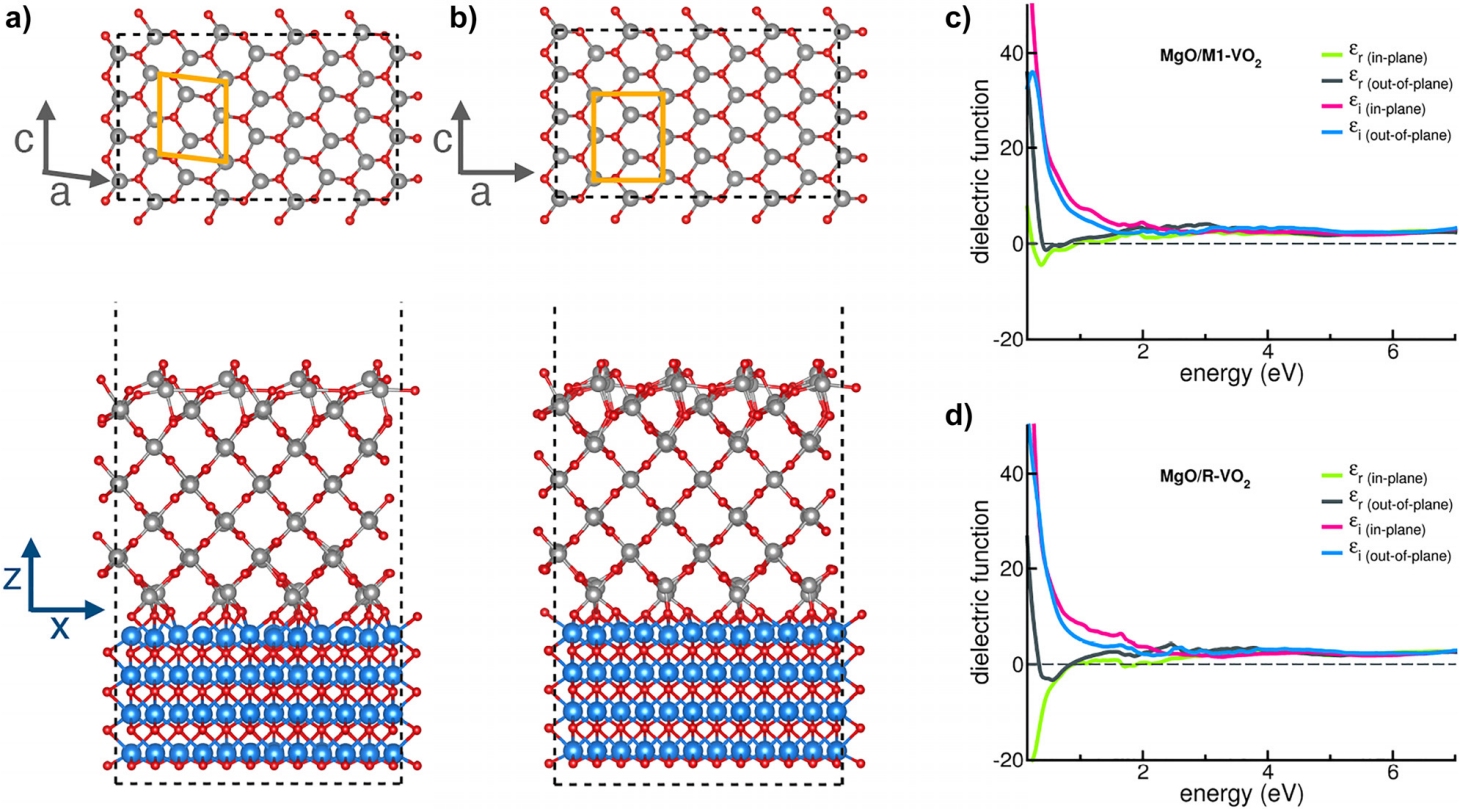
Top and side views of equilibrium geometry of MgO(111)/VO_2_(010) interface with (a) rutile and (b) monoclinic phases, respectively. Dashed lines mark the simulation cell of the interfaces, while the yellow boxes (gray arrows) on top panels identify the unit cell (crystal directions) of the rutile and monoclinic lattices, as in Figure 1. Blue arrows in bottom panel refers to the Cartesian axes of the simulation cell. Real and imaginary parts of the complex dielectric function of the MgO/VO_2_ interfaces for (c) the monoclinic and (d) rutile phases. The in-plane and out-of-plane contributions are explicitly considered.

Even though not directly detectable from optical measurements, for a full theoretical description of the system we considered the model structure of the interface between the MgO(111) and VO_2_(010), in both insulating and metallic phases, and we compared the results with the X-ray analysis of Section 2. The MgO/VO_2_ interfaces are simulated by using periodic supercells of dimension (18.22 × 10.79 × 40.00)Å^3^. The hetero-structures are composed of a slab of MgO(111) with a (2 × 3) lateral periodicity and 0.9 nm of thickness. A 1 nm-thick layer of R-VO_2_(010)-(4 × 4) or M1-VO_2_(010)-(4 × 4) has been deposited on the MgO, to model the interfaces. The lateral periodicity of VO_2_(010) has been chosen to simultaneously best fit the MgO substrate and to host in both the rutile and monoclinic phases. This implied an in-plane uniform strain of the VO_2_ layer (∼2.95%) to match the MgO substrate. Within this geometry, the optical *c*-axis of VO_2_ is aligned along the *y*-axis of the simulation cell (Figure 5), the *a*-axis lies in the *xy* plane and the *b*-axis is oriented out-of-plane, along the *z* axis of the cell. A 1.5 nm vacuum layer is included in the cell to avoid spurious interaction among replica in the direction perpendicular to the surface.

The atomic structures have been fully optimized, through a total-energy-and-forces minimization step, until forces on single atoms were lower than 0.03 eV/Å. The resulting interfaces are shown in Figure 5. In both cases the outermost layers of VO_2_ interact with the substrate forming mixed Mg–O–V bonds at the interface. The rest of the system slightly modifies its structure, except for the top layer, where the external O-atoms readjust to saturate the surface dangling bonds. During the relaxation, the monoclinic state reduced the *β* angle between the original *a* and *c*-axes to accommodate the initial in-plane strain. Nonetheless, the optimized model well reproduces the two VO_2_ phases even in the presence of the MgO substrate. The different electronic character of the M1 and R structures imparts different electronic properties and band alignments at the interface, as shown in the density of states (DOS) plots, reported in Figure S4 of the SI. In the case of the monoclinic structure, the interaction with the substrate has two main effects: (i) a reduction of the VO_2_ bandgap, and (ii) the formation of a type-I band-alignment with the VO_2_ gap fully included in the MgO one. A hybrid MgO/VO_2_ peak appears at the Fermi level (E_*F*_), due to Mg–O–V bonding formation at the interface. This, along with the midgap position of the Fermi energy makes the MgO substrate optically active even at sub-gap energies. Even though a similar mixed orbital peak is present also for the rutile layer, in the case of MgO(111)/R-VO_2_(010) interface the Fermi level is pinned at the top of the MgO valence band, preventing MgO interband transitions for energies smaller than the bandgap (i.e., the substrate remains fully transparent). The different electronic behaviors of the interfaces have an evident effect also on the optical properties, as shown in panels (c) and (d) of Figure 5 that display the real (*ϵ*_*r*_) and imaginary (*ϵ*_*i*_) part of the dielectric function in the directions parallel (in-plane) and perpendicular (out-of-plane) to the interface. In the case of MgO(111)/M1-VO_2_(010) the system has an overall dielectric behavior as for the two separate components (i.e., MgO, and monoclinic VO_2_). The residual negative behavior of *ϵ*_*r*_ at very low energies derives from the excitation of the hybrid bonding states at the Fermi level, which allows for the excitation of low-energy interband transitions, in agreement also with the corresponding imaginary part. Completely different is the case of the rutile VO_2_ interface, which exhibits a metal-like character, with the real part of the dielectric function that diverges to negative values at low energies with a typical Drude-like behavior. This confirms that, even for the explicit simulation of the optical properties of the overall interface, the rutile and monoclinic phases maintain their metal-like and dielectric-like behavior, respectively, as in the bulk. In particular, neither the interaction with the substrate, nor the relaxation of the in-plane strain within the supercell were able to remove the dielectric character of the monoclinic phase.

However, with respect to the experimental measurements, which involve thick structures for both oxide films, the model interfaces simulated here only concern ultra-thin layers for which the interface effects would have been particularly relevant. This means that for these ultra-thin interfaces the simple effective medium approximation does not hold anymore. This, for instance, is the reason why there are strong differences between simulated refractive index and extinction coefficient of the overall interfaces (see Figure S5, SI) and the experimental spectra of Figure 3. In particular, the IR contributions for the interfaces at *λ* > 750 nm in the simulated spectra mostly derives from interband transitions from surface and interface states. In the experimental systems, these effects play a marginal role and can be neglected as demonstrated above.

## 5 Discussion

Even though the anisotropic character of VO_2_ bulk and microcrystals is known for decades [19, 29], the proofs that anisotropy could be maintained also in monolithically integrated thin films are still very scarce [30]. Here, we demonstrated a robust method to monolithically integrate strongly anisotropic VO_2_ thin films. Importantly, this integration is not limited to MgO (111), considered a nonstandard substrate, but can easily be extended to other oxide layers, which support similar epitaxial growth [21]. Several future applications can be envisioned, such as flat optics for beam shaping and sorting, modulators and dichroic optics, but also polarization-sensitive photodetectors [31], not to mention the numerous exotic effects leveraging optical anisotropy for unconventional optical modes, as described in the introduction.

One relevant feature to mention is that the optical absorption of VO_2_ is strongly increased in the metallic state. This should not be seen as a limitation for potential applications but rather as an additional possibility in terms of design. Indeed, switching materials from insulating to absorbing has been recently exploited to expand the design possibilities for nanophotonics, with potential applications for photodetectors, optical limiters, plasmonic switches, optical wavefront shaping and beam-steering [23, 32], [33], [34], [35], [36], [37], [38], [39], [40], [41], [42], [43]. Moreover, the dynamic control of an adjustable negative permittivity within anisotropic thin films could be exploited in exotic physical phenomena that were not experimentally demonstrated yet, such as plasmon canalization and tunneling [44], anisotropy-induced chiral discrimination of surface plasmon [45], or transition from Dirac points to exceptional points [46]. We may also envision an alternative platform for the recently demonstrated quantum simulations in birefringent photonic lattices with tailored losses [47].

**Figure 6:**
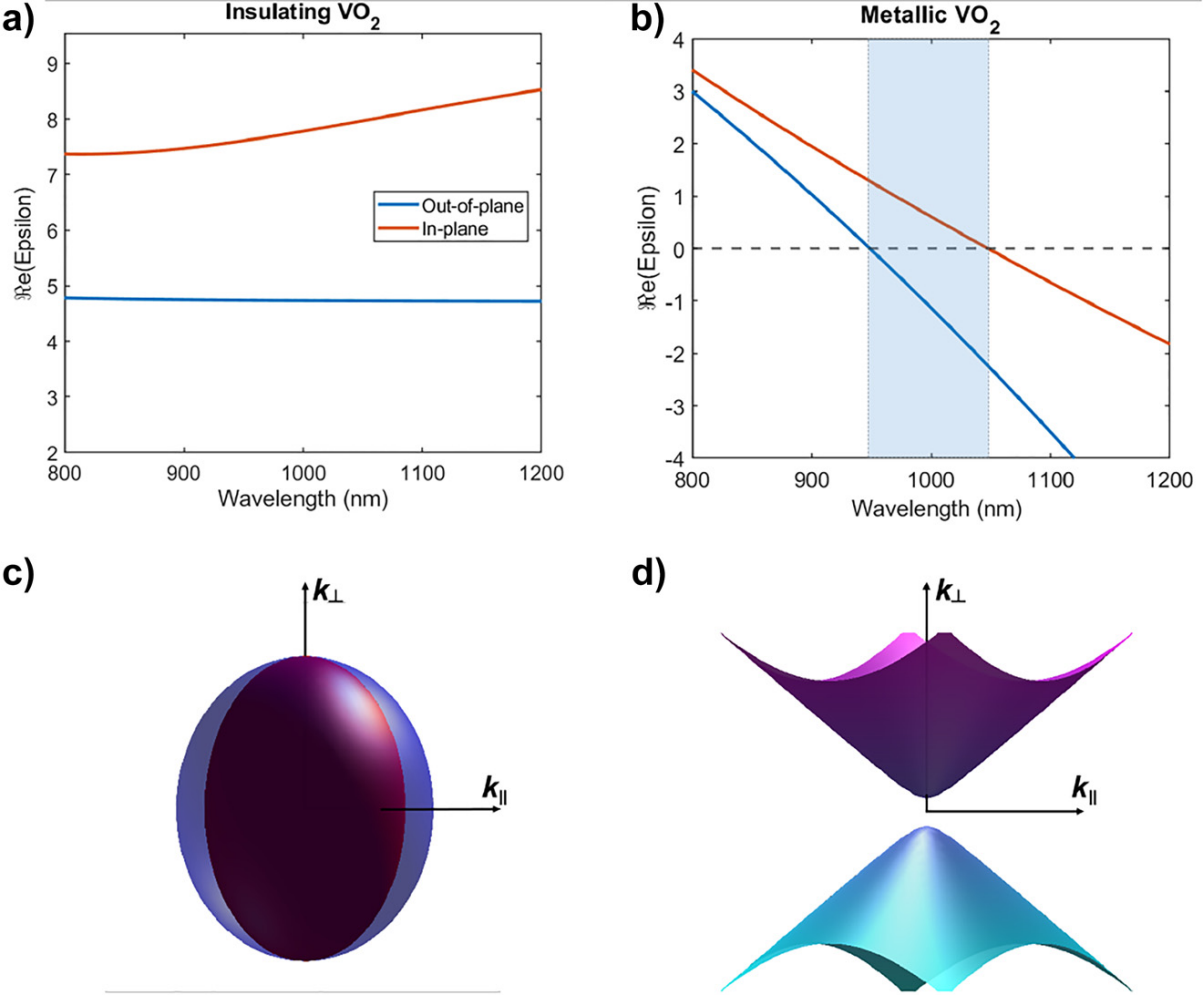
Real part of the permittivity of (a) insulating and (b) metallic VO_2_ in the near-infrared range. This modulation of permittivity results in optical properties changing from birefringent to hyperbolic throughout the range highlighted in shaded blue in (b). (c and d) show the isofrequency contours of insulating and metallic VO_2_, respectively, at *λ* = 1 μm.

Another major outcome of this work appears when we plot the in-plane and out-of-plane components of the real part of the permittivity in both states of VO_2_. As displayed in Figure 6, upon the insulator-to-metal transition the real part of the permittivity becomes negative but not at the same wavelength for the in-plane and out-of-plane permittivities. This results in a 100-nm-wide wavelength range in which the permittivities along different directions have opposite signs. Such an extreme anisotropy, usually named hyperbolic dispersion, is known to produce unconventional optical properties linked to the creation of optical modes with very large wavevectors [48–51].

Therefore, we experimentally demonstrated that epitaxially-grown VO_2_ thin-films can be dynamically tuned from strongly birefringent to hyperbolic in the range 950–1050 nm, as displayed in Figure 6(c and d). Aside from exciting exotic physics such as hyperbolic shear polaritons in monoclinic thin-films [52], the hyperbolic dispersion may find applications in strongly enhanced light–matter interactions [53], hyperlenses [54] or super-Planckian thermal emission [55].

## 6 Conclusions

In summary, we have theoretically and experimentally demonstrated the possibility to grow VO_2_ thin-films integrated in a commensurate way with metal-oxide substrates, such as MgO. The resulting VO_2_ films exhibit the capability to structurally switch between the monoclinic to the rutile phase, as a function of the temperature, as previously known for the corresponding crystalline bulk. This goes in parallel with the possibility to modulate the optical anisotropy of the systems in different spatial directions (e.g., in-plane and out-of-plane), giving rise to a giant birefringence in the IR range. Finally, we revealed that the insulator-to-metal transition of VO_2_ can be exploited to tune the optical anisotropy of the films from birefringent to hyperbolic dispersion. This unique combination of optical characteristics makes VO_2_ the system of choice for the development of an unprecedented set of novel optical applications, from unconventional nanophotonics using boundaries between anisotropic and isotropic media, to polarization-sensitive photodetectors as well as anisotropic systems with a tunable optical loss channel that may be exploited in complex non-Hermitian nanophotonic systems.

## Supplementary Material

Supplementary Material Details
