# Supplementary material for: Tunable optical anisotropy in epitaxial phase-change VO2 thin films
Source: Nanophotonics. 2022 Jun 1;11(17):3913–22. doi: 10.1515/nanoph-2022-0153 (PMC11501475; doi:10.1515/nanoph-2022-0153)
Supplement: Supplementary file 1 — Supplementary Material Details [file j_nanoph-2022-0153_suppl.pdf]

Supporting Information for: Tunable Optical Anisotropy in Epitaxial Phase-Change VO_2 Thin-Films

Jimmy John[1], Amine Slassi[2], Jianing Sun[3], Yifei Sun[4], Romain Bachelet[1], José Pénuelas[1], Guillaume Saint-Girons[1], Régis Orobtchouk[1], Shriram Ramanathan[4], Arrigo Calzolari[2], and Sébastien Cueff[1*]

[1] *Université de Lyon, Institut des Nanotechnologies de Lyon (INL) UMR 5270 CNRS, École Centrale de Lyon, 36 avenue Guy de Collongue, 69134, Ecully, France*

[2] *CNR-NANO Istituto Nanoscienze, I-41125 Modena, Italy*

[3] *J. A. Woollam, Co., 645 M Street, Suite 102, Lincoln, NE 68508, USA and*

[4] *School of Materials Engineering, Purdue University, West Lafayette, IN 47907, USA*

(Dated: May 10, 2022)

I. COMPUTATIONAL DETAILS

All calculations were carried out with the Quantum-Espresso simulation package[1] within the framework of density functional theory (DFT), by using the PBE [2] generalized gradient approximation (GGA) to describe the exchange-correlation (XC) functional. Single particle wavefunctions were expanded in a planewave basis set up to an energy cut-off of 80 Ry; atomic potentials for each chemical species are described by means of Optimized Norm-Conserving Vanderbilt (ONCV) pseudopotentials [3]. A uniform ($2\times$ 4) k-point grid was used for summations over the 2D Brillouin zone. The band gap underestimation due to the standard GGA XC was corrected by including a Hubbard-like potential (U) on both 3d orbitals of vanadium and 2p orbitals of oxygen and 3s orbitals of Mg. The optimized values for the compounds were obtained by means of the pseudohybrid Hubbard Density Functional approach (namely ACBN0) [4]. The effects of the ACBN0 approach on the electronic [5], optical [6] and vibrational [7] properties of several metal oxides have been largely tested in previous reports. The U values resulting from the ACBN0 cycle and used in calculations are: for MgO U(Mg_s)=0.52 eV, U(O_p)=8.30 eV; for VO_2 (rutile) U(V_d)=2.00 eV, U(O_p)=6.93 eV; for VO_2 (monoclinic) U(V_d)=0.95 eV, U(O_p)=6.79 eV.

II. ISOLATED BULK SYSTEMS

Figures S1-S3 summarize the main electronic and optical properties of the separated subsystems of the interfaces, discussed in the main text, namely MgO, R-VO_2 and M1-VO_2. Each Figure indicate (a) the atomic structure; (b) the band structure (left), the total and projected density of states (right); the real (c) and the imaginary (d) part of the complex dielectric function $\hat{\epsilon}(E) = \epsilon_r + i\epsilon_i$, as a function of the energy E of the incoming radiation. Horizontal dashed lines in panels (b) identify the Fermi energy E_F of the system, assumed as zero energy reference. Labels (a,b,c) in panels (c) and (d) refer to the crystal axis directions, as defined in the main text.

* sebastien.cueff@cnrs.fr

Rutile VO_2

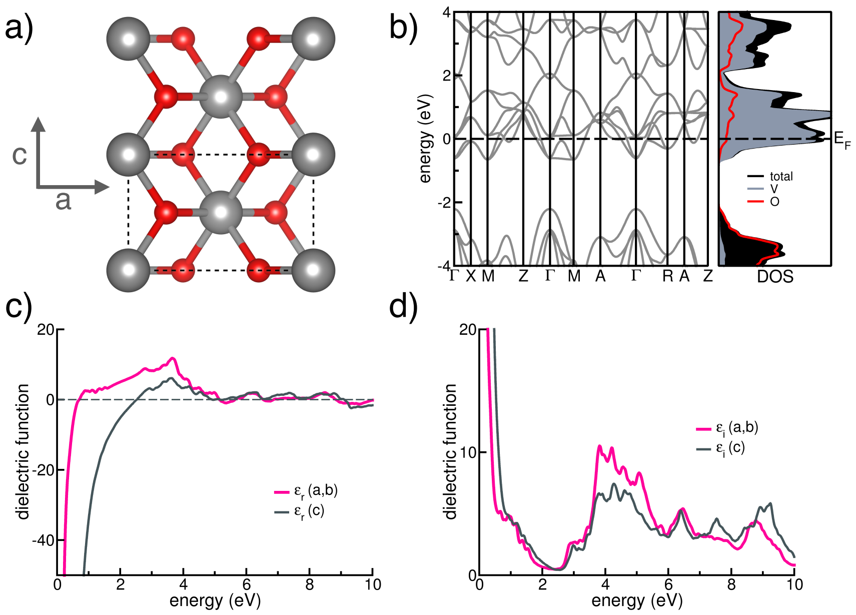

FIG. S1: R-VO_2. a) Atomic structure; b) bandstructure (left panel) and DOS (right panel) plots; real c) and imaginary d) part of the dielectric function along the main crystalline axis.

Monoclinic VO_2

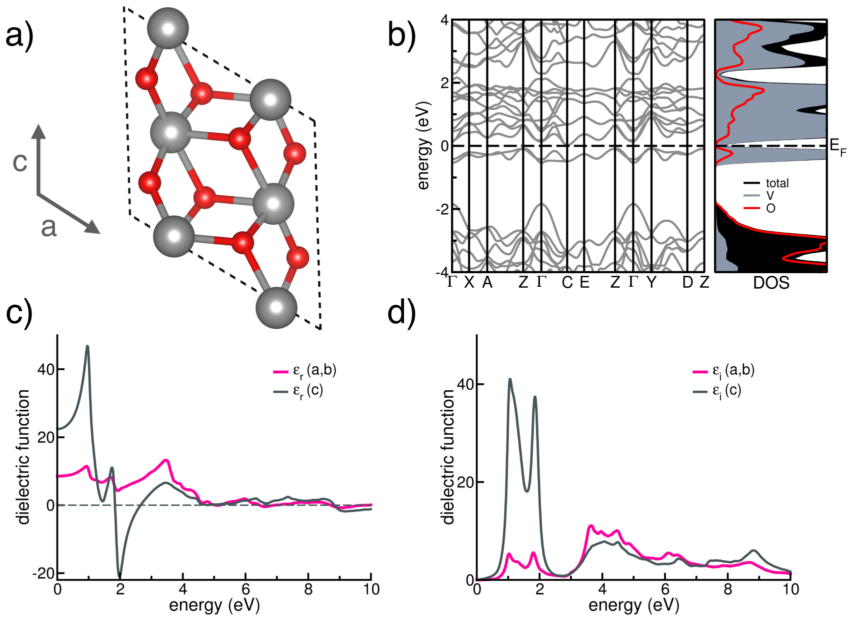

FIG. S2: M1-VO_2. a) Atomic structure; b) bandstructure (left panel) and DOS (right panel) plots; real c) and imaginary d) part of the dielectric function along the main crystalline axis.

Rocksalt MgO

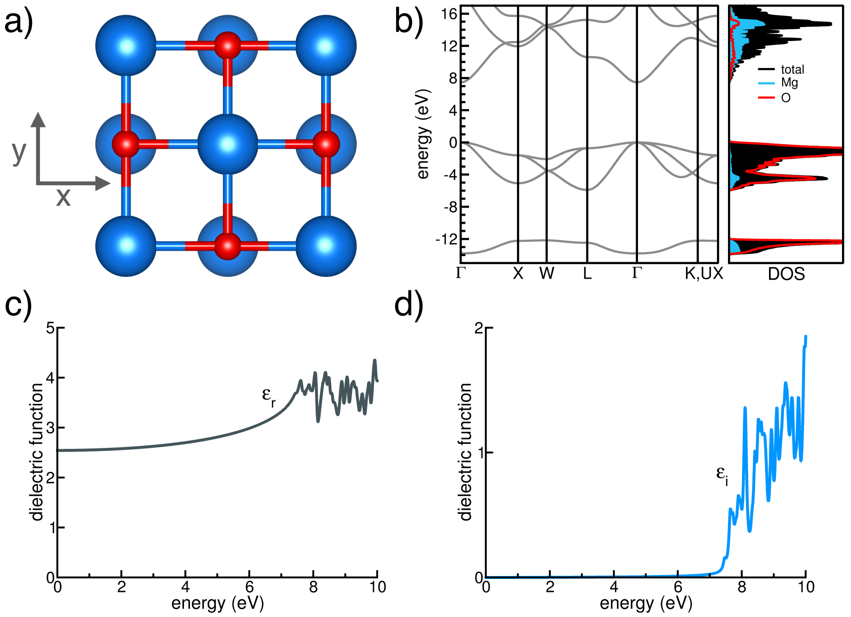

FIG. S3: MgO. a) Atomic structure; b) bandstructure (left panel) and DOS (right panel) plots; real c) and imaginary d) part of the dielectric function along the main crystalline axis.

III. MGO/VO_2 INTERFACES

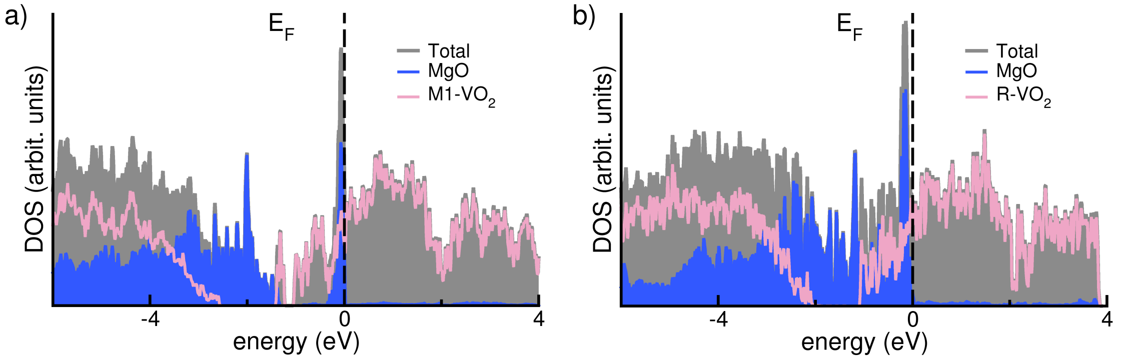

FIG. S4: Total (gray), and MgO- (blue area) and VO_2-projected (pink line) density of states (DOS) of (a) MgO/M1-VO_2 and MgO/R-VO_2 interfaces. Vertical dashed lines mark the resulting Fermi level E_F of the interface, assumed as zero energy reference.

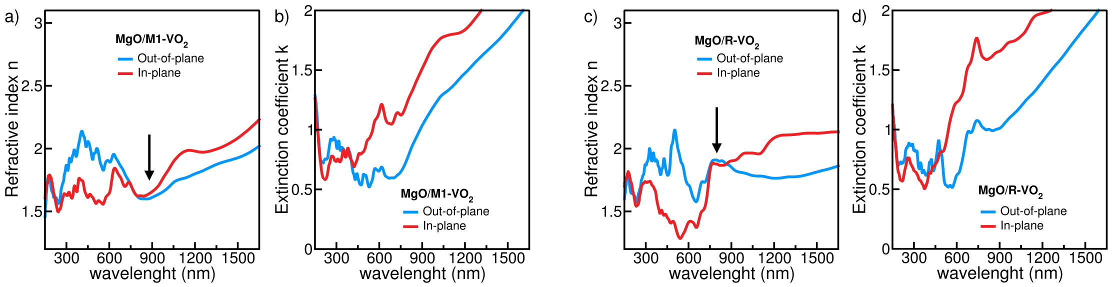

FIG. S5: Simulated refractive index n (a,c) and extinction coefficient k (b,d) along the out-of-plane (blue line) and in-plane (red line) directions for the MgO-VO_2 interfaces, with VO_2 in the monoclinic (M1) and rutile (R) phase, as a function of the wavelength of incoming radiation. The vertical arrows marks the main differences, due to interband transitions involving the MgO substrate.

IV. SPECTROSCOPIC ELLIPSOMETRY MEASUREMENTS AND FITS

Spectroscopic ellipsometry is an optical technique used to measure the spectral amplitude $\tan(\psi)$ and phase (Δ) of the reflected light, which are related to the optical properties and microstructure of the sample under study. The enabling principle of ellipsometry is that p- and s- polarized light reflect differently.

$$R_p \neq R_s$$

With ellipsometry, we measure the complex reflectivity ratio of p- and s- polarized light and reports the result generally in ellipsometric units ψ and Δ,

$$\frac{R_p}{R_s} = tan(\psi)e^{i\Delta} = \rho$$

where $tan(\psi)$ is the magnitude of the ratio of Fresnel reflection coefficients and Δ is the phase difference. Spectroscopic ellipsometry therefore measures the change of phase and polarization of light, which enables the determination of the sample's complex dielectric function (ϵ).

The first step towards analysis of anisotropic properties of VO_2 on MgO, we performed Classic spectroscopic ellipsometry at both VO_2 phases. The optical response is collected in terms of ellipsometric parameters Psi and Delta (ψ and Δ), which can be transformed into material constants "n" and "k" (or equivalently, the complex dielectric functions values "ϵ_1" and "ϵ_2").

Ellipsometric characterization of the sample begins by constructing layered optical model which represents the sample under study. A model is a description of how the data will be "fitted" by the ellipsometry software, in this case Woollam's CompleteEase, to determine the film properties such as thickness and its optical constants. In order to optically characterize the VO_2 sample on MgO, a 3-layer model was used as shown in figure S6. An analytical representation of the different optical responses is needed to correlate the nanofilm structure with its anisotropic optical properties. In our case, our model is composed of three layers: The top layer which is defined as 'layer 2' is the surface roughness layer (VO_2 and air) expressed by an effective medium approximation (EMA) with Bruggeman formula [8].

The middle layer, defined as "layer 1", is the VO_2 that is under study. We use a general oscillator (Gen-Osc) because it models the absorption characteristics by describing the shape

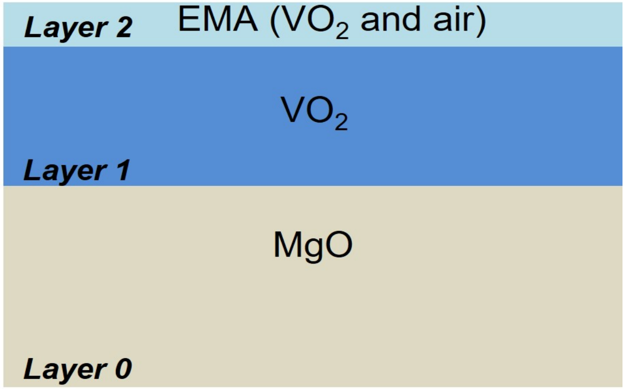

FIG. S6: Layer model used to fit the optical properties of VO_2.

of the absorption properties of the VO_2. It also allows a combination of multiple oscillator line shapes that can be used to represent the optical constants of the material. In our case, in order to model the optical constants of VO_2 at insulator state we use a combination of 4 Gaussian oscillators. This oscillator produces a Gaussian line shape in ϵ_2 with Kramers-Kronig consistent line shape of ϵ_1. Each of these corresponds to interband transitions in the VO_2 sharing a common band gap.

The Gaussian operator represents the complex dielectric function contribution of the oscillator as a function of photon energy (E) in eV, and is expressed as follows:

$\epsilon_{gaussian} = A_n(\Gamma(Z^-) + \Gamma(Z^+) + i[exp(-\Gamma(Z^-)^2) - exp(-\Gamma(Z^+)^2)])$

where

$$Z^+ = \frac{E + E_n}{\sigma_n}$$

and

$$Z^- = \frac{E - E_n}{\sigma_n}$$

While,

$$\sigma_n = \frac{Br_n}{2\sqrt{ln2}}$$

E_n is the centre energy of the oscillator in eV, Br_n is the broadening of the oscillator in eV, A_n is the amplitude of the oscillator, i is the imaginary unit, Γ is a convergence series that produces a Kramer-Kronig consistent line shape for the ϵ_1 dielectric function [9]. Z^+ and Z^- are equivalent to Gaussian Z values, σ is Gaussian standard deviation and n represents total number of oscillators. In our case n is 4. A_n approximately equals ϵ_2 at its

peak value (E_n). By defining σ_n, Br_n approximately equals the full width half maximum value [10].

```
Layer#2 = EMA Thickness #2 = 17.74 nm (fit)

Layer#1 = Biaxial Thickness #1 = 62.82 nm (fit)
Type = Uniaxial
Optical Constants: Difference Mode = OFF

Ex = Gen-Osc

e1 Components
Ein f= 0.00
UV Pole Amp. = 310.3145 (fit)       UV Pole En. 15.000
IR Pole Amp. = 0.000

e2 Components
Oscillator Menu:
Fit Menu : All    None  Amp.  Br.   En.
1 : Type = Gaussian     Amp1 = 0.867888 (fit)   Br1 = 0.3463 (fit)      En1 = 2.917 (fit)
2 : Type = Gaussian     Amp2 = 4.339932 (fit)   Br2 = 1.1707 (fit)      En2 = 3.472 (fit)
3 : Type = Gaussian     Amp3 = 6.216653 (fit)   Br3 = 6.8830 (fit)      En3 = 5.822 (fit)
4 : Type = Gaussian     Amp4 = 1.566537 (fit)   Br4 = 0.8355 (fit)      En4 = 1.110 (fit)

Ez = Gen-Osc

e1 Components
Einf = 0.00
UV Pole Amp. = 255.5651 (fit)       UV Pole En. = 15.000
IR Pole Amp. = 0.000

e2 Components

Oscillator Menu:

Fit Menu : All    None  Amp.  Br.   En.

1 : Type = Gaussian     Amp1 = 3,775254 (fit)   Br1 = 0.3942 (fit)      En1 = 2.670 (fit)
2 : Type = Gaussian     Amp2 = 3.007518 (fit)   Br2 = 1.7145 (fit)      En2 = 3.888 (fit)
3 : Type = Gaussian     Amp3 = 12.627395 (fit)  Br3 = 16.2981 (fit)     En3 = 1.467 (fit)
4 : Type = Gaussian     Amp4 = 1.947377 (fit)   Br4 = 1.3150 (fit)      En4 = 1.162 (fit)

Euler Angles: Phi = 0.00 Theta = 0.00

Substrate Layer #0 MgF2 RC2 Substrate Thickness = 0.5000 mm
```

FIG. S7: VO_2 25°C optical model fit parameters data.

Furthermore, in order to study the anisotropic behaviours of the above VO_2 model, it is possible to convert the VO_2 layer into an anisotropic one, in which the general oscillator layer will be integrated into this new layer. In this layer, it is possible to choose uniaxial anisotropy or biaxial anisotropy. We collected ellipsometry data at different sample orientations rotating around the sample normal. No differences were observed in the raw data, indicating no in-plane anisotropy. In the reported analysis, the optical properties were analyzed as uniaxially anisotropy, with epsilon X = epsilon Y (in plane) and not equal to epsilon Z (out of plane).

```
Layer #2 = EMA Thickness # 2 = 17.90 nm (fit)

Layer #1= Biaxial Thickness #1 = 56.53 nm (fit)
Type = Uniaxial
Optical Constants: Difference Mode = OFF

Ex = Gen-Osc

e1 Components
Einf = 1.000
UV Pole Amp. = 337.0459 (fit)            UV Pole En. = 15.000
IR Pole Amp = 0.000

e2 Components
Oscillator Menu :
Fit Menu : All    None  Amp.  Br.   En.
1 : Type = Gaussian     Amp1 = 7.258018 (fit)   Br1 = 3.5358 (fit)      En1 = 5.438 (fit)
2 : Type = Lorentz      Amp2 = 0.835189 (fit)   Br2 = 0.6702 (fit)      En2 = 2.761 (fit)
3 : Type = Drude (RT)   Resistivity (Ohm cm)3= 0.00034471 (fit)         Scat. Time (fs)3 = 0.820 (fit)
4 : Type = Gaussian     Amp4 = 5.542862 (fit)   Br4 = 1.2737 (fit)      En4 = 3.204 (fit)

Ez = Gen-Osc

e1 Components
Einf = 1.000
UV Pole Amp. = 393.6801 (fit)            UV Pole En. = 15.000
IR Pole Amp. = 0.000

e2 Components
Oscillator Menu:
Fit Menu : All    None  Amp.  Br.   En.
1 : Type = Gaussian     Amp1 = 6.050616 (fit)   Br1=4.8722 (fit)  En1 3.591 (fit)
2 : Type = Lorentz      Amp2 = 1.043808 (fit)   Br2 = 0.3801 (fit)      En2 = 2.548 (fit)
3 : Type = Drude (RT)   Resistivity (Ohm cm)3= 8.7294E-05 (fit)         Scat. Time (fs)3 = 2.979 (fit)
4 : Type = Gaussian     Amp4 = 5.401806 (fit)   Br4 = 0.9857 (fit)      En4 = 3.042 (fit)

Euler Angles: Phi = 0.00      Theta = 0.00

Substrate Layer # 0 MgF2 RC2 Substrate Thickness = 0.5000 mm
```

FIG. S8: VO_2 100°C optical model fit parameters data.

The measured birefringence is between the short and long axis. In our case, each direction of uniaxial anisotropy is described by an individual layer each seeded with 4 Gaussian functions. Therefore, our VO_2 layer will now be expressed as a uniaxial layer with a general oscillator of multiple Gaussian functions for two directions Ex and Ez respectively (fig: S7 and S8). By fitting the data model, it is now possible to extract the uniaxial optical constants of the layer separately. The data set of each is given in S7:

As we see in the above data set, the real part of the optical constants is given by the components e1. It includes four parameters which are E_{inf}, the amplitude of the UV pole, the energy of the UV pole and the amplitude of the IR pole [10].

E_{inf} is a purely real constant added to ϵ_1. It represents the value of ϵ_1 at infinity, which takes into account the effects of absorption well beyond the measured spectral range. While the UV Amplitude and Energy pole describe all the absorption that occur at higher energies outside the measured spectral range. As such, they only affect the real part of the dielectric function ϵ_1. A pole is defined mathematically by :

$$\epsilon_{uv-pole}(E) = \frac{A_n}{E_n^2 - E^2}$$

IR pole amplitude describes the lower energy absorption that are measured outside the spectral range.

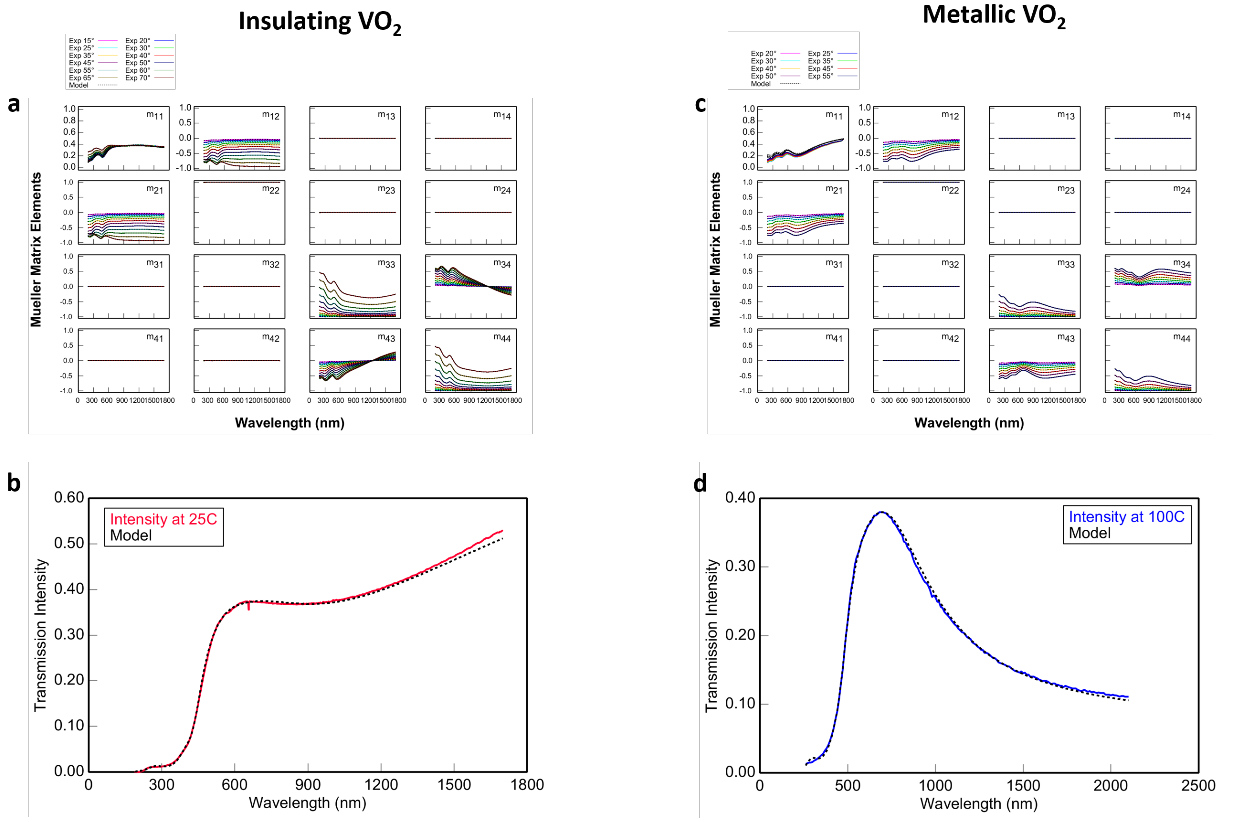

FIG. S9: Optical measurements. a,b) Mueller matrix and transmission measurements for insulating VO_2, measured at room temperature; c,d) Mueller matrix and transmission measurements for metallic VO_2, measured at 100^oC. Solid lines are raw data, dashed lines are fits.

The imaginary part of the data set is represented by e2 components which includes the multiple oscillators. As mentioned before, we use a combination of the 4 Gaussian oscillators

to define the contribution of the imaginary constants ϵ_2. We can observe the fitting values of the different absorption peak points and their associated properties on the data set.

Similarly, to model the VO_2 in its metallic state, we use the same steps as above, except for the oscillator functions. Unlike the previous method, for the metallic VO_2, we use a general oscillator consisting of a combination of Gaussian, Lorentz, and Drude oscillator functions. It is done to incorporate the metallic behaviour of the VO_2 and the changes in their interband absorption properties. Drude model describes the effects of free carriers on the dielectric response, in our case we use Drude RT oscillator as it has resistivity (ρ in Ω.cm) and mean scattering time (τ) as fit parameters. Of which the data set is given as shown S8.

And the final layer is substrate (MgO) and is defined as 'layer 0'. Refractive indices of MgO were pre-determined from a bare substrate and was used for the fits above.

All collected data and fits are shown in Fig. S9, showing the full Mueller matrix as well as the transmission data. We also show as examples the *Rs* and *Rp* measurements on the VO_2 thin films as a function of temperature in Figs. S10 and S11

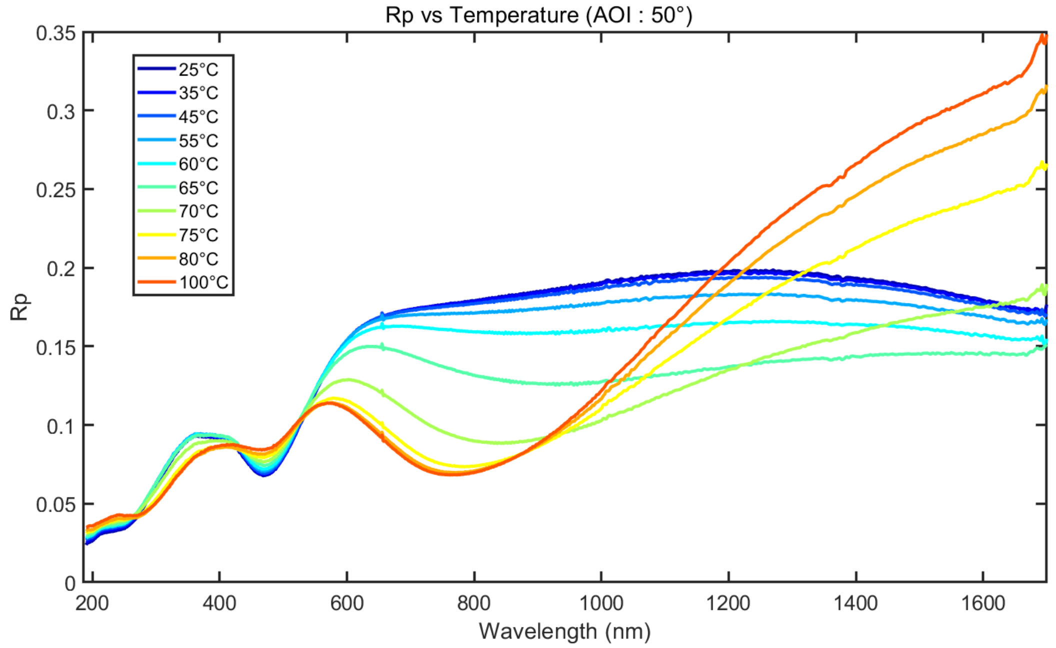

FIG. S10: P-polarized reflection measurements of the VO_2 thin-film as a function of temperature (angle of incidence: 50°)

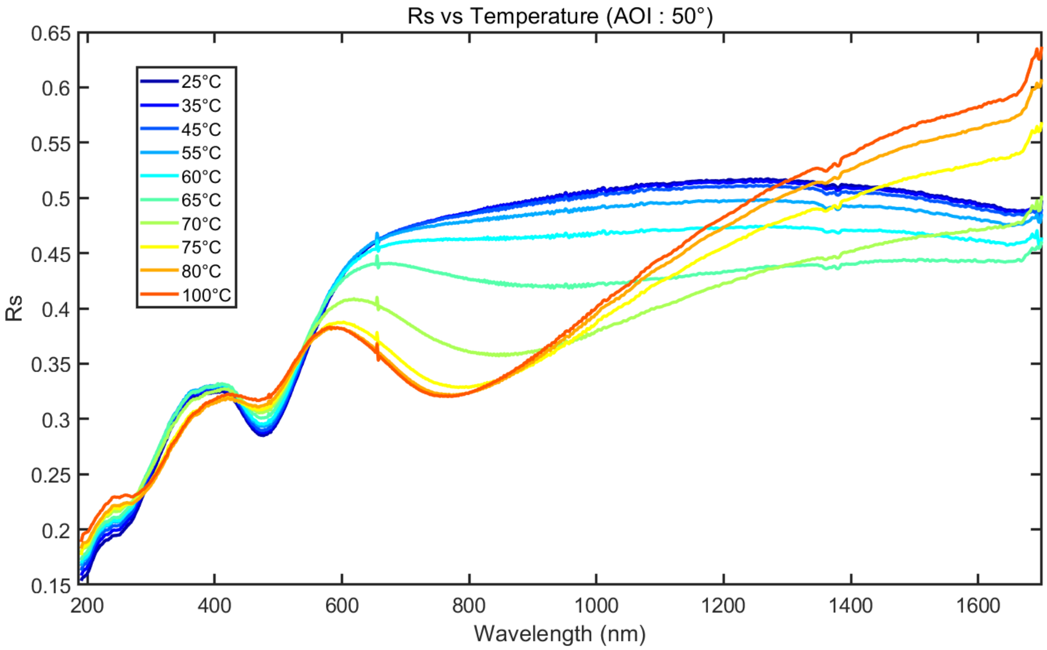

FIG. S11: S-polarized reflection measurements of the VO_2 thin-film as a function of temperature (angle of incidence: 50°)

---

[1] P. Giannozzi, O. Andreussi, T. Brumme, O. Bunau, M. Buongiorno Nardelli, M. Calandra, R. Car, C. Cavazzoni, D. Ceresoli, M. Cococcioni, N. Colonna, I. Carnimeo, A. D. Corso, S. de Gironcoli, P. Delugas, R. A. DiStasio, A. Ferretti, A. Floris, G. Fratesi, G. Fugallo, R. Gebauer, U. Gerstmann, F. Giustino, T. Gorni, J. Jia, M. Kawamura, H.-Y. Ko, A. Kokalj, E. Küçkbenli, M. Lazzeri, M. Marsili, N. Marzari, F. Mauri, N. L. Nguyen, H.-V. Nguyen, A. O. de-la Roza, L. Paulatto, S. Poncé, D. Rocca, R. Sabatini, B. Santra, M. Schlipf, A. P. Seitsonen, A. Smogunov, I. Timrov, T. Thonhauser, P. Umari, N. Vast, X. Wu, and S. Baroni, Advanced capabilities for materials modelling with Quantum ESPRESSO, J. Phys.: Cond. Matt. **29**, 465901 (2017).

[2] J. P. Perdew, K. Burke, and M. Ernzerhof, Generalized gradient approximation made simple, Phys. Rev. Lett. **77**, 3865 (1996).

[3] D. R. Hamann, Optimized norm-conserving vanderbilt pseudopotentials, Phys. Rev. B **88**, 085117 (2013).

[4] L. A. Agapito, S. Curtarolo, and M. Buongiorno Nardelli, Reformulation of dft+u as a pseudo-hybrid hubbard density functional, Phys. Rev. X **5**, 011006 (2015).

[5] P. Gopal, M. Fornari, S. Curtarolo, L. A. Agapito, L. S. I. Liyanage, and M. Buongiorno Nardelli, Improved predictions of the physical properties of Zn- and Cd-based wide band-gap semiconductors: A validation of the ACBN0 functional, Phys. Rev. B **91**, 245202 (2015).

[6] A. Calzolari, A. Catellani, M. Buongiorno Nardelli, and M. Fornari, Hyperbolic Metamaterials with Extreme Mechanical Hardness, Adv. Opt. Mater. **9**, 2001904 (2021).

[7] A. Calzolari and M. Buongiorno Nardelli, Dielectric properties and Raman spectra of ZnO from a first principles finite-differences/finite-fields approach, Sci. Rep. **3**, 2999 (2013).

[8] H. Fujiwara, J. Koh, P. I. Rovira, and R. W. Collins, Assessment of effective-medium theories in the analysis of nucleation and microscopic surface roughness evolution for semiconductor thin films, Phys. Rev. B **61**, 10832 (2000).

[9] D. D. S. Meneses, M. Malki, and P. Echegut, Structure and lattice dynamics of binary lead silicate glasses investigated by infrared spectroscopy, Journal of non-crystalline solids **352**, 769 (2006).

[10] J. Woollam *et al.*, Completeease data analysis manual, JA Woollam Co. Inc, New England. United States of America (2011).
